# Recreational cannabis legalization and alcohol purchasing: a difference-in-differences analysis

**DOI:** 10.1186/s42238-021-00085-x

**Published:** 2021-07-07

**Authors:** Collin M. Calvert, Darin Erickson

**Affiliations:** School of Public Health, Division of Epidemiology and Community Health, University of Minnesota, 1300 S. 2nd Street, Suite 300, Minneapolis, MN 55454-1015 USA

**Keywords:** Cannabis legalization, Difference-in-differences, Alcohol purchasing, Substitution, Complementarity

## Abstract

**Background:**

Whether recreational cannabis legalization is associated with changes in alcohol consumption (suggesting a potential substitution or complementary relationship) is a key question as cannabis policy evolves, particularly given the adverse health and social effects of alcohol use. Relatively little research has explored this question.

**Methods:**

This study examined the association between recreational cannabis legalization and alcohol purchasing in the USA using an interrupted time series design. We used data from the Nielsen Consumer Panel (2004–2017) from 69,761 households in all 50 states to calculate monthly milliliters of pure ethanol purchased for four beverage categories (beer, wine, spirits, and all alcohol products). We used difference-in-differences models and robust cluster standard errors to compare changes in milliliters of pure ethanol purchased. We fit models for each beverage category, comparing three “policy” states that have legalized recreational cannabis (Colorado, Oregon, and Washington) to states that had not legalized recreational cannabis. In one set of models, a single control state was selected that matched pre-policy purchasing trends in the policy states. In another set, policy states were compared to all states that had not legalized recreational cannabis.

**Results:**

Compared to all other states that did not legalize recreational cannabis, Colorado households showed a 13% average monthly decrease in purchases of all alcoholic products combined (estimate, 0.87; CI, 0.77, 0.98) and a 6% decrease in wine (0.94; CI, 0.89, 0.99). Estimates in Washington were suggestive of an increase in spirits purchased in both the unrestricted (1.24; CI, 1.12, 1.37) and restricted sample (1.18; CI, 1.02, 1.36). Oregon showed a significant decrease in monthly spirits purchased when compared to its selected comparator state (0.87; CI, 0.77, 0.99) and to all other states without legalized recreational cannabis (0.85; CI, 0.77, 0.95).

**Conclusions:**

Results suggest that alcohol and cannabis are not clearly substitutes nor complements to one-another. Future studies should examine additional states as more time passes and more post-legalization data becomes available, use cannabis purchase data and consider additional methods for control selection in quasi-experimental studies.

## Background

Alcohol consumption remains a major public health problem in the USA, increasing risk of fatal traffic crashes, heart disease, and several types of cancer (Klatsky et al. 2015; Room et al. 2005; Zaloshnja et al. 2013). According to 2018 data from the National Survey on Drug Use and Health (NSDUH), over 55% of people report being current drinkers (i.e., consumed alcohol in the past 30 days) (Alcohol Facts and Statistics 2019). Drinking in the USA is increasing over time. A recent meta-analysis found annual increases in both alcohol use (0.30% per year [95% CI, 0.22%, 0.38%]) and binge drinking (0.72% per year [95% CI, 0.46%, 0.98%]) from 2000 to 2016 (Grucza et al. 2018). Martinez and colleagues found increases of per-capita alcohol sold for beer, spirits, and wine products from 2003 to 2016 in the USA nationally and across the majority of states (Martinez et al. 2019). This trend in increased alcohol consumption raises concerns for alcohol-related morbidity and mortality.

One factor that may affect alcohol consumption is the use of products that act as either substitutes or complements. These products are likely to be other intoxicating substances like cannabis (Crost and Guerrero 2012). Two drugs are considered substitutes if the use of one increases as use of the other decreases, and are considered complements if the use of both increases in tandem (Hursh and Roma 2016). Interventions affecting one substance can thus affect others, such as policies that increase or decrease availability or cost of one substance may cause increased or decreased use of the other substance. When considering policies that increase or decrease the use of harmful substances like alcohol, it is important to keep in mind what effects the policies may have on consumption of other intoxicating and potentially harmful substances. The potential substitute or complement to alcohol that we examine in this study is cannabis.

A growing body of research has begun to examine how cannabis legalization relates to alcohol purchasing and consumption (Lucas et al. 2013; Moore 2010). A literature review cannabis and alcohol articles found a mixture of studies supporting substitution, complementarity, or no relationship (Subbaraman 2016). Subbaraman noted, however, that results tended to differ depending on the study population and the study design; cross-sectional studies often supported complementarity while three-quarters of longitudinal studies supported substitution. This review was later replicated and expanded to include animal studies, and authors found a similar mixture of studies supporting complementarity, substitution, or no relationship (Risso et al. 2020). In another literature review, focused specifically on cannabis policies and alcohol consumption, there was a dearth of studies examining recreational cannabis legalization and alcohol (Guttmannova et al. 2016). Notably, the authors recommend that future studies consider a difference-in-difference approach when examining how cannabis legalization and alcohol may be associated at the state level given its ability to account for unmeasured characteristics and relative dearth among prior studies. Salomonsen-Sautel et al. used Fatality Analysis Reporting System (FARS) data to examine fatal alcohol-involved traffic crashes as they related to medical cannabis legalization. Comparing Colorado to 34 control states, they found no evidence of a change in crashes post-legalization of cannabis (Salomonsen-Sautel et al. 2014). In contrast, a study of NSDUH data comparing states with legalized medical cannabis to states that had not legalized medical cannabis, found that binge drinking among adults of legal drinking age increased 6–9% after medical cannabis legalization (Wen et al. 2014). More recently, two studies have looked at changes in alcohol sales and use following recreational cannabis legalization. Using state tax receipt data, Veligati et al. found no relationship between legalization and alcohol sales across all 50 states (Veligati et al. 2020). In Washington between 2014 and 2016, no significant changes were seen in self-reported alcohol consumption; however, self-reported alcohol-related harms decreased post-legalization (Subbaraman and Kerr 2020). Using data from the National Survey on Drug Use and Health (2004–2017), poly use of alcohol and cannabis increased while the use of alcohol alone decreased following recreational cannabis legalization (Kim et al. 2021). In summary, there is yet no consensus on the nature of the possible relationship between alcohol and cannabis consumption. In addition, the degree to which changes in alcohol consumption after legalization of recreational cannabis differs depending on the type of alcoholic beverage (e.g., beer, spirits) has not been explored.

The goal of this paper is to determine whether legalization of recreational cannabis is associated with changes in alcohol purchasing (a proxy for consumption). In addition, we measure this association for individual alcoholic beverage types (beer, spirits, and wine). No studies have examined different beverage types, nor studied this question using purchasing data from the Nielsen Consumer Panel. We use monthly purchasing time series data (2004–2017) to model changes in alcohol by volume purchased by households in several states that legalized recreational cannabis (Colorado, Washington, and Oregon) compared to states that did not legalize. These three policy states were selected given they had sufficient post-legalization data for analyses. While more recent studies have begun examining how cannabis legalization relates to alcohol use through quasi-experimental methods such as ours, none have explored this relationship using the Nielsen Consumer Panel (which offers a larger sample size through household-level rather than aggregate state-level data). Given the lack of consensus among studies of the relationship between alcohol and cannabis, more research is needed. Our study builds off of previous work and extends it by examining this question across different types of alcoholic beverages.

## Methods

### Design

**Fig. 1:**
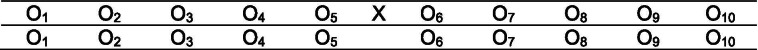
Controlled interrupted time series study design. Each O represents an observed outcome value in the time series, and X represents the “interruption” when the policy took effect. The top series is considered the treated series while the bottom series is the no-treatment control series

We used a quasi-experimental controlled interrupted time series (CITS) design (Lopez Bernal et al. 2017, 2019). Quasi-experiments are designs similar to randomized controlled trials, but without random allocation to treatment of control groups. Despite this, quasi-experimental designs (particularly the interrupted time series, or ITS) tend to have higher internal validity than observational studies given the exposures or treatments are exogenous (Biglan et al. 2000; Shadish et al. 2002). Including a control group further strengthens internal validity by accounting for history effects, while having longitudinal data helps addresses maturation bias (Bonell et al. 2011). CITS is an extension of both ITS and DiD, combining the benefits of both by making use of multiple timepoints before and after an intervention (ITS) and incorporating a comparison group that mimics the treated group’s counterfactual (DiD) (Lopez Bernal et al. 2017, 2019). One significant advantage of the CITS design is that non-time varying household factors (both measured and unmeasured) are accounted for (Lopez Bernal et al. 2017). In our study, time series for control units are included to model the counterfactual outcome of the treated unit in the absence of recreational cannabis legalization. Figure 1, adapted from Cook and Campbell (1979), illustrates this design.

Colorado, Oregon, and Washington were designated as policy states. A single control state was selected and paired with each policy state to improve internal validity. Control states were selected through graphical examination of purchasing trends and the strength of the Pearson correlation coefficient for purchasing between the policy state and control state pre-legalization. In the unrestricted sample, New Jersey served as a control for Colorado, Texas for Washington, and Virginia for Oregon. Because the trend in alcohol purchasing changed in the restricted sample, different control states were selected for the restricted sample; North Carolina was a control for Colorado, Illinois for Washington, and West Virginia for Oregon. In addition, we fit models in which all non-policy states (i.e., states that within the timeframe of this study had not legalized recreational cannabis) were treated as controls.

### Data sources

Data were obtained from the Nielsen Consumer Panel, a dataset of U.S. households across all 50 states that provide information on their household demographics and the products they purchase. Participating households use in-home scanners to track all of their purchases, where and when they make purchases, and how much they pay for each product. Participants are randomly sampled proportionately based on county population, and are balanced across several household characteristics (e.g., income, education level). In addition, Nielsen provides sampling weights to project its sample to national, regional, and market area levels. Approximately 80% of participating households remains in the panel from 1 year to the next. Each year, the consumer panel consists of between 40,000 and 60,000 households. Using data from 2004 to 2017, two samples were created: a unrestricted sample of households (hereafter referred to as the “unrestricted sample”), including those that may have left the panel prior to legalization or joined following legalization; and a sub-sample, hereafter referred to as the “restricted sample,” that was restricted to households with data both prior to and following legalization. This restricted sample was analyzed because some households may drop out or enters the Nielsen Consumer Panel immediately following legalization (and thus would not contribute data pre-policy or post-policy, respectively). Prior studies on this topic have employed a range of designs (including cross-sectional), however, having data on the same households over a period of time may be better for measuring substitution/complementarity (Subbaraman 2016). Our unrestricted sample consisted of 178,232 individual households across the USA with varying amount of years spent as part of the panel. The restricted sample consisted of 69,761 individual households.

### Measures

#### Alcohol purchasing

Data from the Nielsen Consumer Panel dataset includes information on individual alcohol products purchased and the volume of each individual beverage. A previous validation study of the quantity purchased measurements in the Nielsen Consumer Panel found them to match with sales record data 94% of the time (Einav et al. 2010). We used these data to construct measures of monthly alcohol purchased by each household. To calculate our outcomes, we multiplied the number of beverages purchased by the volume of the product, separately for beer, wine, and spirits (spirits are also referred to as liquor, or alcoholic beverages with higher alcohol content than wine or beer). We then multiplied this by a static proportion that represented the average amount of ethanol in a product for each type of alcoholic beverage. These values (0.05 for beer, 0.40 for liquor, and 0.13 for wine) are based on the American Epidemiologic Data System, where the average proportion of ethanol is 0.045 for beer, 0.411 for liquor, and 0.129 for wine (Doernberg and Stinson 1985). We then aggregated across month and state to create a measure of monthly pure ethanol purchased for each state in milliliters. We also constructed three additional outcome variables for specific types of alcoholic beverages. These outcomes were the pure ethanol purchased of beer, of wine, and of spirits. Because distributions of ethanol purchased across all three beverage types were skewed, we applied a natural logarithmic transformation to each. Estimates were then exponentiated to back-transform them for ease of interpretation.

#### Legalization of recreational cannabis

We included binary variables that indicated legalization of recreational cannabis for the three policy states: Colorado, Washington, and Oregon. These variables were coded as a 0 before the date of legalization, and a 1 on and after the date of legalization. For example, legalized recreational cannabis in Colorado went into effect on December 10, 2012. The indicator for legalization in Colorado was coded as a “0” for January 2004 through November 2012, and a “1” for December 2012 through December 2017. In a difference-in-difference model, this represents the “time” variable. We also created an indicator variable for “policy” states versus “control” states, which serve as the “treated” variable. Colorado, Washington, and Oregon were coded as a “1” while all other states, which served as potential comparators for the controls, were coded as a “0.”

#### Household characteristics

Four of the household measures recorded in the Nielsen Consumer Panel were included to adjust for any imbalances between policy and control states: household income, household size, marital status, and race. These characteristics were selected based on their expected impact on alcohol purchasing both prior to and following legalization of recreational cannabis, while also being likely unaffected by the policy change itself. Substance use and purchasing can vary across levels of income and social inequality (e.g., structural racism) (Bailey et al. 2017; D. R. Williams and Mohammed 2013). Thus, we included household income levels and race (a proxy for racism) in our models. Marital status serves as a proxy for social support. Marital status has shown to be an important predictor of alcohol use (Leonard and Rothbard 1999). The size of the household was included to account for households with multiple adult residents who purchase alcohol, and differences between households with multiple purchasers versus households with a single resident. Household income was measured by 11 categories (ranging from < $5,000 per year to over $200,000). Household size measured the number of individuals living in the household in 9 categories (from 1 to 9 or more). Marital status was measured as whether the heads of household were married, widowed, divorced/separated, or single. Race was measured as whether the household was primarily White, Black, Asian, or a different racial identity.

### Analysis

First, we calculated descriptive statistics of the households in our analytic sample from January 2004 to December 2017. Each of the measures is presented as weighted averages by incorporating the frequency weights that were included in the Nielsen Consumer Panel. These weights are updated each year and correct for selection bias in sampling of households. The sum of these weights is equal to the total number of U.S. households.

To estimate the relationship between recreational cannabis legalization and alcohol purchasing over time, we used fixed effects linear regression models with an interaction term for legalization of recreational cannabis (binary) and policy/control state (binary) as the test of our primary hypothesis, commonly referred to as difference-in-difference models. This modeling approach was chosen over random effects (or “mixed models”); random effects models offer no important benefits over fixed effects in this context but are vulnerable to violation of the random effects assumption (and consequently produce biased effect estimates). The basic structure of our DiD models is illustrated below:

$${Y}_{ijt}={\beta }_{0}+{\beta }_{1}{Treated}_{j}+{\beta }_{2}{Policy}_{t}+{\beta }_{3}{Treated}_{j}\mathrm{*}{Policy}_{t}+{\beta }_{4}{V}_{ijt}+{\varepsilon }_{ijt}$$

*Y*_*ijt*_ represents the outcome for household *i* in state *j* at time *t*; *Treated*_*j*_ is an indicator for whether or not a state ever legalized recreational cannabis; *Policy*_*t*_ is an indicator for the time *t* when recreational cannabis has been legalized (this value matches between for households in policy and control states in each model); *V*_*ijt*_ is a vector of household-level time-varying covariates. The coefficient *β*_*3*_ for the interaction term between *Treated* and *Policy* is the effect estimate of interest and represents the change in alcohol purchasing before versus after recreational cannabis legalization in a given policy state compared to the control state (or states).

We fit separate models for each policy state compared to a matched no-policy state, and models for each policy state compared to all no-policy states. We did this for both the unrestricted sample of households as well as the restricted sample (restricted to households with observations prior to and following legalization). We fit separate models for each policy state because policy implementation dates were staggered (i.e., occurred on different dates); including the three policy states together in one model would induce bias in regression estimates (Goodman-Bacon 2018). Fixed effects for time were included to account for seasonality and time trends. Standard errors were adjusted for clustering at the household level using an extension of the Huber-White sandwich estimator to generate robust cluster standard errors. This can be accomplished using the vce(cluster) option in Stata (Williams 2000). All analyses were done using Stata version 16.

## Results

### Sample characteristics

The characteristics of households in the unrestricted sample within each policy state are shown in Table 1. Overall, states had similar distributions of household size, marital status, and race. A majority of households had annual incomes of $45,000-$124,999, were comprised of 2–3 residents, had heads-of-household who were married, and primarily identified as white. Average monthly alcohol purchased for all alcoholic beverages combined by a given household was highest in Washington (415 mL per month) and lowest in Colorado (230 mL per month). In the unrestricted sample of households, alcohol purchased was higher in Washington compared to its matched control, but similar in Colorado and Oregon compared to their respective controls. When comparing alcohol purchased in the unrestricted sample to the restricted sample, Colorado and Oregon showed noticeable changes in purchasing trends prior to legalization; from a decreasing trend to a flat or minor increasing trend in Colorado, and from a decreasing trend to an increasing trend in Oregon.

**Table 1:** Sociodemographic characteristics of study sample in three states with legalized recreational cannabis

	Colorado (*n* = 3619)	Washington (*n* = 4444)	Oregon (*n* = 2380)
Household income			
< $5000	1%	2%	2%
$5000-$7999	2%	2%	2%
$8000-$14,999	5%	6%	10%
$15,000-$29,999	15%	17%	20%
$30,000-$44,999	15%	17%	17%
$45,000-$69,999	22%	23%	21%
$70,000-$99,999	20%	18%	16%
$100,000-$124,999	16%	14%	11%
$125,000-$149,999	< 1%	< 1%	< 1%
$150,000-$199,999	1%	< 1%	< 1%
$200,000 +	< 1%	< 1%	< 1%
Household size			
1 person	27%	27%	26%
2 people	34%	33%	34%
3 people	16%	15%	17%
4 people	14%	14%	13%
5 people	5%	7%	7%
6 people	2%	2%	3%
7 people	1%	1%	< 1%
8 people	< 1%	< 1%	< 1%
9 or more people	< 1%	< 1%	< 1%
Marital status			
Married	51%	50%	52%
Widowed	9%	10%	8%
Divorced/separated	19%	20%	21%
Single	21%	20%	20%
Race			
White	85%	83%	89%
Black	4%	3%	1%
Asian	2%	6%	3%
Not White, Black, or Asian	9%	8%	7%
Milliliters of pure ethanol mean (standard deviation)			
All alcoholic beverages	230 (876)	415 (1131)	379 (1124)
Beer	84 (440)	125 (547)	111 (504)
Spirits	104 (591)	156 (665)	132 (698)
Wine	42 (267)	134 (592)	135 (596)

Statistics presented in the above table are from the unrestricted sample of households (i.e., not limited to households with data before and after legalization) using Nielsen frequency weights. Data are from 2004 to 2017. Household income is the self-reported income of a household upon entry into the NCP. Household size is the number of people living in a household. Race refers to the self-reported racial identity of the household. Milliliters of pure ethanol is the average milliliters of ethanol purchased by a given household in a given month

### Recreational cannabis legalization and alcohol purchasing

Table 2 presents the estimates for monthly changes in alcohol purchased in each policy state compared to controls for the unrestricted sample, adjusting for household characteristics. Estimates are back-transformed from natural-logarithmic values for interpretability, to represent the percent change in milliliters of alcohol purchased (i.e., (1-beta)*100. Among the unrestricted sample, legalization of recreational cannabis in Colorado was associated with a 13% decrease in purchasing for all alcohol products combined (beta, 0.87; CI, 0.77, 0.98) and a 6% decrease in purchasing for wine products (0.94; 0.89, 0.99) when compared to all non-policy states. In Washington, legalization was associated with a 24% increase in purchase of spirits (1.24; 1.12, 1.37) but a 12% decrease in purchase of wine (0.88; 0.79, 0.98) when compared to its single control state. These associations remained statistically significant when compared to all non-policy states. Oregon, when compared to a single control state as well as all non-policy states, saw statistically significant reductions in spirits purchased post-legalization.

**Table 2:** Monthly changes in alcohol purchasing (95% CI) per household by beverage category: policy states vs. control states

	Unrestricted sample		Restricted sample	
	Single control state e^Beta^ (95% CI)	All non-policy states e^Beta^ (95% CI)	Single control state e^Beta^ (95% CI)	All non-policy states e^Beta^ (95% CI)
Colorado	(*n* = 7978)	(*n* = 171,829)	(*n* = 2843)	(*n* = 46,461)
All alcoholic products	0.90 (0.77, 1.06)	**0.87 (0.77, 0.98)**	0.92 (0.74, 1.14)	0.94 (0.80, 1.09)
Beer	0.93 (0.82, 1.05)	0.95 (0.87, 1.05)	0.96 (0.81, 1.13)	1.01 (0.90, 1.13)
Spirits	0.98 (0.88, 1.09)	0.96 (0.88, 1.04)	0.95 (0.82, 1.11)	0.97 (0.86, 1.08)
Wine	0.94 (0.86, 1.02)	**0.94 (0.89, 0.99)**	1.01 (0.90, 1.14)	0.99 (0.92, 1.06)
Washington	(*n* = 18,641)	(*n* = 172,694)	(*n* = 3556)	(*n* = 46,673)
All alcoholic products	1.07 (0.92, 1.25)	1.11 (0.97, 1.27)	1.08 (0.89, 1.31)	1.11 (0.94, 1.30)
Beer	1.05 (0.94, 1.18)	1.06 (0.96, 1.16)	1.04 (0.91, 1.19)	1.03 (0.92, 1.15)
Spirits	**1.24 (1.12, 1.37)**	**1.25 (1.14, 1.37)**	**1.18 (1.02, 1.36)**	**1.21 (1.07, 1.36)**
Wine	**0.88 (0.79, 0.98)**	**0.91 (0.83, 1.00)**	0.91 (0.80, 1.03)	0.94 (0.84, 1.05)
Oregon	(*n* = 6680)	(*n* = 170,736)	(*n* = 1095)	(*n* = 56,871)
All alcoholic products	1.06 (0.85, 1.34)	0.97 (0.81, 1.18)	0.97 (0.71, 1.33)	1.08 (0.86, 1.35)
Beer	1.05 (0.88, 1.25)	1.03 (0.89, 1.18)	1.01 (0.78, 1.32)	1.12 (0.94, 1.32)
Spirits	**0.87 (0.77, 0.99)**	**0.85 (0.77, 0.95)**	0.94 (0.79, 1.13)	0.96 (0.84, 1.09)
Wine	1.03 (0.88, 1.20)	0.97 (0.85, 1.11)	1.05 (0.87, 1.28)	1.03 (0.88, 1.21)

Models are fixed effects linear regression models with robust cluster standard errors, adjusted for household income, household size, marital status, and race. In each model, n is equal to the number of clusters (i.e., households) included for both the policy state and selected control(s). Estimates are exponentiated from log-transformed values to represent the average percent change in mean monthly alcohol purchasing per household following legalization of recreational cannabis. For example, a value of 0.87 corresponds to a 13% decrease while 1.25 corresponds to a 25% increase. The unrestricted sample is all households from 2004 to 2017. The restricted sample is limited to households from 2004 to 2017 that had data both before and after legalization of recreational cannabis in a given policy state. “Single control state” refers to models where a given policy state was compared to a single, matched control state. “All non-policy states” refers to models where controls were all states that did not legalize recreational cannabis. Bolded values represent *p* values < 0.05

Results from models fit to the restricted sample of households are also shown in Table 2. All estimates that were significant in the unrestricted sample became non-significant in the restricted sample with the exception of spirits in Washington. In Washington, legalization of recreational cannabis was associated with an 18% increase in spirit purchasing when compared to a single control state (1.18; 1.02, 1.36) and a 21% increase when compared with all non-policy states (1.21; 1.07, 1.36).

## Discussion

The present study builds on prior literature by measuring the association between recreational cannabis legalization and alcohol purchasing among a longitudinal, nationally representative sample of households. We analyzed both the unrestricted sample of households as well as a restricted sample that consisted only of households with purchasing data prior to and following legalization of recreational cannabis. Across all beverage types, we found only a few significant associations between legalization and purchasing. Across both the unrestricted sample and the restricted sample, the only significant estimate was an increase in spirit purchasing associated with cannabis legalization in Washington. Nonetheless, the largely non-significant findings are consistent with several prior studies that have examined alcohol-related outcomes and cannabis legalization (Salomonsen-Sautel et al. 2014; Subbaraman and Kerr 2020; Veligati et al. 2020).

One reason for largely non-significant findings may be the sample of households contained within the Nielsen Consumer Panel, which do not explicitly capture purchases made by subgroups (e.g., age, race) and tend to oversample higher-income households. Despite the inclusion of frequency weights to correct for the latter issue, these are unlikely to be perfect. In addition, the relationship between alcohol and cannabis likely varies depending on subgroup. In a meta-analysis of 39 studies of alcohol/cannabis substitution and complementarity, Subbaraman found an almost equal number of articles supporting substitution, complementary, and independence (Subbaraman 2016). However, studies of youth participants (i.e., under 21) tended toward substitution. Saffer analyzed data from the National Household Survey of Drug Abuse (NHSDA) and found that white and black respondents were more likely to complement with alcohol following cannabis decriminalization while Hispanic respondents were more likely to substitute (Saffer and Chaloupka 1999). Particularly for findings from the restricted sample, estimates may not be statistically significant due to a reduction in power (the number of households was reduced when limiting the datasets to households that had data both preceding and following legalization of recreational marijuana). This loss of power may partially explain why estimates that were statistically significant in the unrestricted sample were non-significant in the restricted sample.

Other societal-level factors, such as policies that affect alcohol purchasing and use, may have biased results despite the inclusion of control states. For instance, the state of Washington approved Initiative 1183 to allow the privatization of spirit sales (formerly, only state liquor stores were legally allowed to sell spirits) on March 1, 2012. Consequently, private stores were allowed to begin selling spirits while all state-run stores closed by May 31, 2012. Because this change occurred around the same time as the legalization of recreational cannabis, it may not be possible in the present analysis to disentangle the effects of cannabis legalization from the effects of alcohol privatization. However, previous studies of the effects privatization in Washington had on spirit sales have shown little to no change in alcohol consumption following the shift to privatization (Kerr et al. 2018; Ye and Kerr 2016).

Whether alcohol and cannabis are substitutes or complements for one another may depend on the type of alcoholic beverage. The only consistent statistically significant result in the present study was in purchases of spirits in Washington, with spirits having a complementary relationship to cannabis. Prior studies have also shown the potential for substitution or complementarity between alcohol and cannabis depending on beverage type. A study by Clements and Daryal using cross-price elasticities found that alcohol may be a weak substitute for cannabis, but that the strength changes depending on beverage: 0.5 for spirits, 0.2 for wine, and 0.1 for beer (higher numbers imply a stronger substitution relationship) (Clements and Daryal 2005). Miller and Plant found that teenagers in the UK were more likely to use illicit drugs (e.g., cannabis) if they consumed higher amounts of beer or spirits compared to wine, suggesting a complementary relationship between illicit drugs and spirits (as well as beer) (Miller and Plant 2003). Similarly, in a study of English adolescents, participants who drank spirits were more likely to use cannabis (complementary relationship) and other illicit drugs (Sutherland and Willner 1998).

## Limitations

Our findings must be considered in light of several limitations. Randomization of those exposed to and unexposed to the policy was not possible, so states were selected as controls based on their comparability to each policy state. These controls represent counterfactuals for each policy state, modeling what would have happened in a given policy state had recreational cannabis not been legalized. However, because there is no perfect control in a quasi-experimental study, the controls are considered non-equivalent. In addition, confounding bias cannot be fully accounted for despite these efforts and the inclusion of several key covariates (e.g., households’ income). Thus, residual confounding may still bias our estimates. Some of the threats to internal validity that come with a non-equivalent control group, such as maturation or regression bias, are mitigated by incorporating a long pre-policy time series and conditioning on several key household covariates (Shadish et al., 2002). In addition, we selected controls using two criteria: graphical examination of parallel trends, a common strategy used for DiD models (Angrist and Pischke 2008); and the strength of Pearson correlation coefficients for alcohol purchasing prior to cannabis legalization. Future studies may consider other methods for improving the comparability of policy and control states, such as synthetic control matching (Bouttell et al. 2018). Synthetic controls were not used in the present analysis.

With a total of 48 models run, there is a possibility that any statistically significant findings are due to chance (i.e., a type I error). Because of this, statistically significant effect estimates must be interpreted with caution. Another limitation is the lack of data available on cannabis purchasing and use. The Nielsen Consumer Panel does not currently include data on purchasing for cannabis, so we were unable to directly compare alcohol purchasing between cannabis users and non-users. Instead we used date of cannabis legalization to represent households that had access to (and potentially used) cannabis. Prior studies have used this same method to approximate exposure to cannabis. In addition, data were limited to purchases up to the end of 2017; though several more states than the ones included in the present study have legalized recreational cannabis, there was not sufficient post-legalization data. As data becomes more available, analyses should incorporate additional states. Finally, medical cannabis legalization was not examined. Prior studies have indicated that medicinal and recreational cannabis may differ in how they relate to alcohol use (Lucas et al. 2013) Gunn et al. found that recreational cannabis users tended to drink more alcohol on days they used cannabis when compared to medical cannabis users (Gunn et al. 2019). More studies are also needed that compare how the relationship between alcohol and cannabis may differ by type of cannabis, and by the mode of cannabis use (e.g., smoked, edibles).

Households are able to enter and exit the dataset, meaning households may enter immediately following legalization or may exit immediately preceding. Nielsen notes that recruitment to replace households that drop out is on-going, that efforts are made that recruited households match dropped households as closely as possible, and that the majority of households remain in the panel for multiple years. Regardless, it is possible that households entering or exiting the panel around the time of legalization may have different levels of alcohol purchasing compared to households that remain in the panel. To address this, we analyzed both the entire dataset of households (the “unrestricted sample”) and a limited dataset of only households that were present both before and following legalization.

## Conclusions

Overall, findings do not provide strong evidence of a relationship between recreational cannabis legalization and alcohol purchasing, but further research is needed with additional states and methods for modeling quasi-experimental data before stronger conclusions can be made. Alcohol may substitute or complement cannabis depending on subgroup characteristics, including any history of substance abuse or age. Results may be informative to states considering whether or not to legalize recreational cannabis, or states concerned about the unintended effects on use of harmful substances (e.g., alcohol). As cannabis becomes legalized and more widely available across the USA, there is a greater need to understand any unintentional consequences these policy changes may have for alcohol-related harms and public health problems more broadly. Our findings do not suggest a significant change in alcohol purchasing after legalization of recreational cannabis.

## Data Availability

The dataset analyzed for this study is available for purchase through the James M. Kilts Center for Marketing: https://www.chicagobooth.edu/research/kilts.

## Abbreviations

NSDUH: National Survey on Drug Use and Health
FARS: Fatality Analysis Reporting System
ITS: Interrupted time series
DiD: Difference-in-difference
